# Supplementary material for: Content-rich biological network constructed by mining PubMed abstracts
Source: BMC Bioinformatics. 2004 Oct 8;5:147. doi: 10.1186/1471-2105-5-147 (PMC528731; doi:10.1186/1471-2105-5-147)
Supplement: Additional File 2 — The original results of the above study (non-essential files are deleted to keep the file size under the limit set by BMC bioinformatics). [file 1471-2105-5-147-S2.bz2 › chilibotAdditionalFile2/dip10/688ID11867639E18007/html/AES_MALT.html]

 


 **AES** and **MALT** 
  
Found 5 abstracts in PubMed, retrieved 5.  
 

 What does Google say? 
 PDF only 
| .edu only 

---

**Interactive relationship** (e.g. stimulation, inhibition, etc)

**Inhibitory relationship**- However, we have identified an amino acid substitution in  **MalT**  that suppresses  **MalT**  inhibition by  **Aes**  without interfering with its inhibition by MalY, suggesting that the binding sites of the two inhibitory proteins do not coincide.  Ref: 11867639 J Biol Chem, 2002
- The expression of plasmid encoded  **aes**  resulted in strong repression of the maltose transport genes in  **malT**  strains 10 fold reduction, but not in a  **malT**  Con strain which is independent of the inducer.  Ref: 9401025 J Bacteriol, 1997
**Neutral relationship**- These mutations are located at positions 3 2 43, and 359, most likely defining the interaction patch with  **Aes**  on the three dimensional structure of  **MalT** .  Ref: 12003949 J Bacteriol, 2002
- In addition, MalK, the ABC component of the maltodextrin transport system, and the two enzymes MalY and  **Aes**  down regulate  **MalT**  activity in vivo.  Ref: 11867639 J Biol Chem, 2002
- The activity of  **MalT**  is depressed through a direct protein protein interaction with  **Aes** .  Ref: 12374803 J Biol Chem, 2002
- The  **Aes**  protein directly controls the activity of  **MalT** , the central transcriptional activator of the Escherichia coli maltose regulon.  Ref: 11867639 J Biol Chem, 2002
- Also, overproduction of  **MalT**  counteracted the  **Aes**  dependent repression, indicating a direct interaction between  **MalT**  and  **Aes** .  Ref: 9401025 J Bacteriol, 1997
- MalK, the ATP hydrolyzing subunit of the cognate ABC transporter, MalY, an enzyme with the activity of a cystathionase, and  **Aes** , an acetyl esterase  [ **AES** ] , phenotypically act as repressors of  **MalT**  activity.  Ref: 12003949 J Bacteriol, 2002

**Non-interactive relationship** (e.g. studied together, co-existance, homology, etc.)

- **Aes** , a 36 kDa acetylesterase from Escherichia coli, belongs to the hormone sensitive lipase family, and it is involved in the regulation of  **MalT** , the transcriptional activator of the maltose regulon.  Ref: 12374803 J Biol Chem, 2002
- The differential effects of ATP and ADP on the competition between the inducer and  **Aes**  or MalY suggest that the ATPase activity displayed by  **MalT**  plays a role in the negative control of its activity.  Ref: 11867639 J Biol Chem, 2002
- By using a biochemical approach, we demonstrate here that i  **Aes**  controls  **MalT**  activity through direct protein protein interaction, ii  **Aes**  competes with maltotriose for  **MalT**  binding, iii ATP and ADP differentially affect the competition between  **Aes**  and the inducer, and iv part, if not all, of the  **Aes**  binding site is located in DT1, the N terminal domain of the activator, which also contains the ATP binding site.  Ref: 11867639 J Biol Chem, 2002
